# Novel therapies for perioperative respiratory complications

**DOI:** 10.15171/jcvtr.2017.21

**Published:** 2017-09-25

**Authors:** Jahan Porhomayon, Leili Pourafkari, Ali El-Solh, Nader D Nader

**Affiliations:** ^1^Clinical Professor of Anesthesiology and Critical Care Medicine, Division of Critical Care Medicine, Buffalo, NY, USA; ^2^State University of New York at Buffalo, Jacobs School of Medicine and Biomedical Sciences, Buffalo, NY, USA; ^3^Cardiovascular Research Center, Tabriz University of Medical Sciences, Tabriz, Iran

**Keywords:** Respiratory, Perioperative, Surgery, Complication

## Abstract

Postoperative respiratory complications are of paramount clinical importance as they prolong the hospitalization, increase the costs of treatment and contribute to the perioperative mortality. Obesity, preexisting pulmonary disease and advanced age are known risk factors for developing postoperative respiratory complications, which affect exceeding number of patients. Hereby, we present a review on the pathogenesis of post-operative respiratory complications particularly in obese and older patients. We further focus on the standard management and emerging therapies for the post-operative respiratory complications.

## Introduction

Obesity, preexisting pulmonary disease and advanced age are among the risk factors for developing postoperative respiratory complications. It is estimated that postoperative respiratory complications can range from 5% to 80% in surgical patients.^1^

Obesity alone or combined with increasing age presents a new set of challenges for the perioperative team. Obesity is a serious problem resulting in major health consequences and is strongly associated with acute and chronic medical conditions.^2^ The rise in the rate of obesity in adults in the last 30 years is a clear indication that obesity crisis needs to be addressed.^3^ Obese patients have propensity to develop respiratory complications such as atelectasis, reduced flow patterns such as decreased expiratory flow, inability to clear secretions, reduced functional residual capacity, and lung volumes. These changes can be much more severe in morbidly obese patients.^4^

At the same time, we have seen an increasingly older population in surgical practices. As a consequence of increase in life expectancy, we can expect physiologic changes in almost every major organ in the body. Older patients have complex hemodynamic needs such as higher blood pressure, lower ejection fraction and reduced oxyhemoglobin levels. A clinician must balance and consider these altered physiologies when assessing patient for surgery.^5^ Additionally, there are other complications related to other postoperative adverse events, such as renal dysfunction, bleeding, electrolyte disturbances and infections.^6, 7^ The introduction of enhanced recovery after surgery (ERAS) concepts has also reduced complications rate. ERAS protocol includes counseling such as: no prolong fasting, no selective bowel preparation, thromboprophylaxis, Mid-thoracic epidural, short acting anesthetics, normothermia and avoidance of salt and water overload, prevention of postoperative nausea and vomiting, early mobilization and nutrition and early catheter removal.^8^ It is therefore obvious to realize that preventing pulmonary complications requires considering preoperative, intraoperative as well as post operatives events. In this review we will focus on the pathogenesis as well as emerging therapies for post-operative respiratory complications particularly in obese and older patients.

### Respiratory alteration in obesity and elderly

The impact of obesity on respiratory system can vary from minimal to significant alteration in gas exchange and lung volumes.^9, 10^ There are multitudes of changes including changes in fat content and its mechanical contribution chest, abdominal and upper airway.^11^ As a result of these mechanical changes, compliance of chest wall and lung are reduced. The expiratory reserve volumes, functional residual capacity and residual volume incrementally decreases as body mass index indices increases. In elderly patients there are anatomical changes related to chest wall and thoracic spine deformity that result in increased work of breathing and decreased respiratory system compliance. Additionally, changes involving lung parenchyma result in expansion of air spaces. The muscles of respirations gets weaker and contribute to impaired gas exchange and poor cough result in atelectasis and further deterioration of oxygenation and ventilation.^5, 12, 13^ The changes in respiratory system are more progressive when obesity is associated with advanced age or in association with overlap syndromes.

### Sleep and respiratory disorders in obese and elderly population

Sleep apnea syndrome (SAS) presents a major challenge for the practicing clinician in the perioperative setting.^14,15^ Respiratory complications are common and could be life threatening.^16^

SAS in the elderly presents with various clinical symptoms and also has many complications.^17^ Older patients are susceptible to developing SAS due to many factors including coexistence of chronic respiratory organ disorders, increase in upper airway collapse, prior strokes, cardiovascular diseases, hypertension, as well as other physiological or anatomical changes that accompany aging.^18^ Chronic obstructive pulmonary disease (COPD) in older obese adults represents a unique challenge to clinicians and involves multiple important changes in elderly population. These changes involve impaired pulmonary function and results in hypoxia and poor response to bronchoconstriction. The elderly patients are at increased risk of complications and mortality after surgery.^19^ The management strategies for COPD patients include pneumococcal and influenza vaccinations and, smoking cessation, as well as the use of short-and long-acting bronchodilators. Intervention is frequently required with oxygen therapy, and occasionally noninvasive positive airway pressure for acutely decompensated states.^19^

### Postoperative respiratory complications of obese and elderly patients

In general, elderly population may experience pulmonary complications such as hypoxic or hyper-carbic respiratory failure, bronchospasm, trachea-bronchitis, pulmonary emboli, pneumothorax, acute lung injury, aspiration, pneumonia or atelectasis after non-cardiac surgery.^20^ Obesity alone increases the risk of pulmonary complications by 4.5 folds or by 310%.^20^

There are surgical as well as medical risk factors contributing to pulmonary complications. Surgical factors related to respiratory complications are more common in major abdominal and thoracic procedures as well as in operations lasting more than 2.5 hours.^21^

Furthermore, there are patient related risk factors that need to be assessed.^22^ They include advanced age, obesity, history of COPD or smoking,^23^ and a forced expiratory rate of 9 seconds.^21^ Some of the patient related risk factors are potentially modifiable. Most obesity surgery centers have protocols to assess and modify risk factors for the optimal patient outcome.

### The management of respiratory complications in the immediate postoperative period

Early detection of respiratory compromise is the key to the successful management of post-operative patients. The process for prevention of respiratory complications starts with the thorough assessment of risk factors for respiratory compromise in post-operative patients. Two important patient related risk factors are obesity, and extreme age, which commonly accompany other comorbidities. Older and obese patients with COPD and SAS are particularly predisposed to respiratory complications.

**Figure 1:**
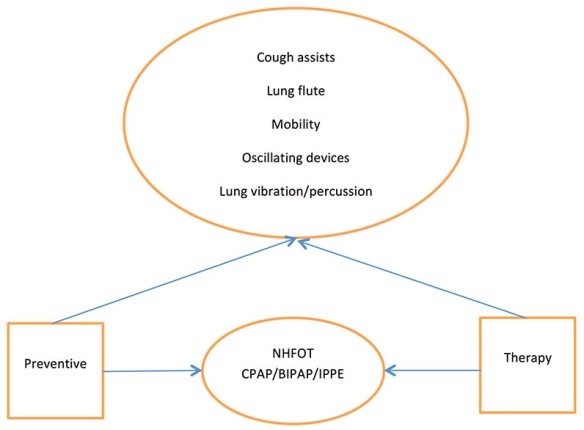
Schematic display of the therapeutic approaches for treatment of tracheobronchial secretions and chest physiotherapy.

Respiratory compromise in post-operative patients is usually preventable with appropriate monitoring and early detections of patients at high risk for such complications. The role of non-invasive ventilation and oxygenation strategies are evolving and must be understood for proper utilization of these technologies. Bi-level positive airway pressure (Bi-PAP), continuous positive airway pressure (CPAP) and nasal high flow oxygen therapy (NHFOT) are frequently used for prevention as well as treatment of acute respiratory failure (Figure 1).

### Emerging technologies in the management of respiratory complications in peri-operative period

The clinical approach to management of post-operative respiratory disorders is multifaceted and is mostly concentrated on a team-based approach.

Manufacturers responsible for respiratory monitoring technologies are putting enormous effort to develop strategies that could improve early detection of respiratory compromise, and most hospitals are actively seeking ways to reduce the risk of respiratory dysfunction in the post-operative period.^24^ Events leading to undesirable respiratory events following surgery/anesthesia^25^ typically initiate with atelectasis, diaphragmatic dysfunction, immobility and inability to clear secretions.^26^ Therefore it is important to detect, reverse and treat contributing factors related to respiratory insufficiency. It is common practice to encourage deep breathing^27^ coughing,^28^ chest physiotherapy,^29^ postural drainage,^30^ aerosol therapy^31^ and institute positive pressure therapy. Several emerging technologies have been proposed that could be utilized in the immediate post-operative period in case conventional therapies are deemed inadequate to reverse the progression of respiratory disorders. NHFOT was introduced to clinical practice in early 2000.^32^ A non-invasive high-flow respiratory support system can be effective in improving oxygenation with humidification^33^ in hypoxic patients. Application of the system in the adult surgical high dependency unit appears appropriate.^34^ NHFOT was used initially in racehorses to meet the extremely high respiratory demands by high flow of oxygen at rates up to 60 liters per minute. It was subsequently patented by Fisher and Pike for clinical use in medicine with similar principle to provide high oxygen flow rate and low positive pressure^35^ to meet the excessive respiratory demand during periods of hypoxia and respiratory compromise.^36^ HFNOT as a novel method of respiratory support in diverse clinical settings has been gaining popularity. It is applied increasingly in critically ill patients and in the perioperative care of the high-risk subset of patients.^37^ HFNOT has been used for several indications that include hypoxemic respiratory failure,^38^ cardiogenic pulmonary edema, counterbalancing auto-positive end expiratory pressure (auto-PEEP) in patients with COPD and as a prophylactic or therapeutic measure in treatment of respiratory failure following operation.^39,40^ It has been shown to be safe and non-inferior to conventional CPAP in providing prophylactic support to very preterm neonates, and adults after extubation.^41,42^ The technology has been validated for use in neonates and small children and is becoming increasingly popular to use in general hospital ward as well as the critical care units. It has the capability to produce low positive pressure^43^ with delivery of high oxygen concentration while maintaining the ability of the patient to have effective cough and clear secretions. The gas flows from 50 Psi wall source to a device where pressure is further reduced to 8 Psi and is delivered via nasal cannula with humidification to the patient. The device is very easy to use and is better tolerated compared to conventional nasal oxygen delivery cannula.

Positive pressure technique^44^ is another modality that is frequently utilized in post anesthesia recovery units to induce sputum production. Positive expiratory technique was approved by FDA for use in patient with COPD and chronic bronchitis but it is increasingly used in patients with lung collapse from secretions or mucous plug in the immediate perioperative period.^31^ Intermittent positive pressure breathing (IPPB) devices uses a respirator for delivery of the controlled -gas volume to assist in expansion of lung and prevents atelectasis, thereby improving patient tidal volumes.^45^ IPPB machines are likewise utilized for the delivery of aerosol medications. IPPB may be useful in patients at risk of respiratory failure resulting from postoperative decreased respiratory function and in patients with acute severe bronchospasm or exacerbated COPD, who do not recover adequately by standard treatments. Cough assist devices in the form of mechanically applied positive pressure breath followed by negative pressure, in order to help stimulate a deep natural cough, have been utilized in the postoperative care units.

**Figure 2:**
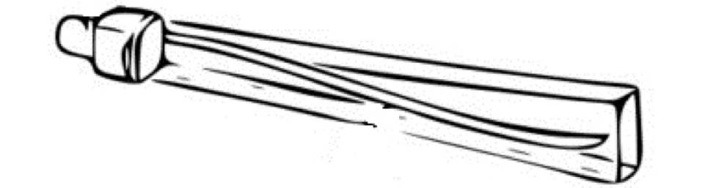
Lung flute.

Lung flute is another vibrating device utilized in clinical practice where a vibration of low frequency audio of approximately 18 Hz generated by flute results in breaking the secretions in the lung and help the patient to have more effective ability to cough up the retained secretions The Lung Flute technology utilize sound waves to break up tracheobronchial secretions (Figure 2).^46,47^ Inside the horn of the Lung Flute, there is a mouthpiece over a reed that is used for expiration. A forceful exhalation into this device causes retrograde travel of the acoustic wave back into the lower airways and lung parenchyma, thereby increasing mucocilliary clearance.^48^ The major limitations of this device is the need for reasonable effort to exhale and small chance of pneumothorax.^49^

Oscillating devices may be used to clear the secretion of the lung through generating oscillations that can be delivered internal or external to the chest wall.^50^ Extra-thoracic oscillations are delivered to the chest wall from outside,^51,52^ while intra-thoracic oscillations are capable of creating adjustable resistance within the airways that mobilizes mucus secretions by producing controlled levels of positive pressure.

**Figure 3:**
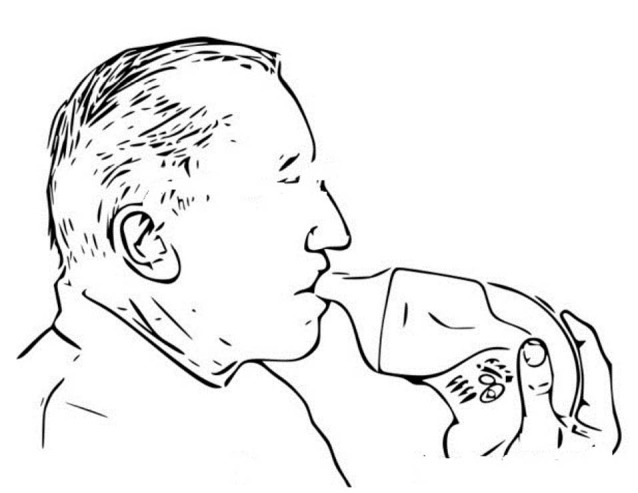
Vibralung Acoustical Percussor, that provides acoustic vibration of gas column in to the tracheobronchial tree with respiration to promote effective sputum production.

Breathing exercises with the application of a device that includes vibration and humidification of inspired air is effective for increasing secretion clearance.^53, 54^ FDA granted clearance to market such devices. In Vibralung Acoustical Percussor, sound waves vibrate a column of gas into the tracheobronchial tract over a wide range of frequencies (5 to 1200 Hz) during inspiration and expiration to promote effective sputum production (Figure 3) (Table 1).

**Table 1:** Techniques applied to help remove secretions from respiratory system

**Methods**	**Characterization**
Mobility/physical therapy	Procedure that depends on patient mobility
Physiotherapy	Devices based on outside chest wall operation, for example tapping, pulsation, and postural drainage management
Positive inspiratory and negative inspiratory	Machine applied positive-pressure, followed by negative pressure breath
Positive inspiratory breath	Positive pressure breath beginning at the airway opening
Positive expiratory pressure	Exhalation against a fixed resistor that creates an increase in airway pressure, like flute and Acupella
Obligatory breath	Directed at open glottis stimulating cough
Incentive spirometry	Active breathing exercises
High frequency chest wall percussion/compression	Surge of air applied through covering worn by patient
Vibration and sound wave	High frequency sound waves with percussion

Speedy mobilization in early postoperative period is currently considered the key to avoid respiratory complications.^55^ Multiple studies have supported the concept of early mobilization.^56, 57^ It is the clinician and institutional responsibility to encourage and develop mobilization protocols in the hospital and acute care settings.^58,59^ Airway clearance devices have potential value as a supplement to mobilization protocols.

In conclusion, postoperative pulmonary complications are of paramount clinical importance: they prolong the hospitalization, increase the costs of treatment and contribute to the perioperative mortality. Early diagnostics is essential and possible only through continuous and meticulous surveillance of the patient. In the postoperative period several factors contribute to the development of the postoperative pulmonary complications: medications, pain, influence of the trauma of the operation, decreased lung capacity and decreased mobilization. Prevention of complications is crucial. The cornerstones of adequate postoperative care are oxygen therapy, sufficient analgesia, and physiotherapy. With the observation of earliest signs of respiratory insufficiency commencement of respiratory support is mandatory. Artificial ventilation or assist devices could be used as preemptive measures to prevent the development of pulmonary complications.^60^

In order to make definitive recommendations for the use of cough assist devices or airway clearance devices, adequately-powered long-term randomized controlled trials are needed.^61^

## Ethical approval

Not applicable.

## Competing interests

The authors declare there is no conflict of interest.
